# Therapeutic Peptides to Treat Myocardial Ischemia-Reperfusion Injury

**DOI:** 10.3389/fcvm.2022.792885

**Published:** 2022-02-17

**Authors:** Carlota Fernandez Rico, Karidia Konate, Emilie Josse, Joël Nargeot, Stéphanie Barrère-Lemaire, Prisca Boisguérin

**Affiliations:** ^1^Institut de Génomique Fonctionnelle, Université de Montpellier, CNRS, INSERM, Montpellier, France; ^2^Laboratory of Excellence Ion Channel Science and Therapeutics, Valbonne, France; ^3^PHYMEDEXP, Université de Montpellier, CNRS, INSERM, Montpellier, France

**Keywords:** myocardial infarction, ischemia-reperfusion injury, therapeutic peptide, pharmacological treatment, cardioprotection

## Abstract

Cardiovascular diseases (CVD) including acute myocardial infarction (AMI) rank first in worldwide mortality and according to the World Health Organization (WHO), they will stay at this rank until 2030. Prompt revascularization of the occluded artery to reperfuse the myocardium is the only recommended treatment (by angioplasty or thrombolysis) to decrease infarct size (IS). However, despite beneficial effects on ischemic lesions, reperfusion leads to ischemia-reperfusion (IR) injury related mainly to apoptosis. Improvement of revascularization techniques and patient care has decreased myocardial infarction (MI) mortality however heart failure (HF) morbidity is increasing, contributing to the cost-intense worldwide HF epidemic. Currently, there is no treatment for reperfusion injury despite promising results in animal models. There is now an obvious need to develop new cardioprotective strategies to decrease morbidity/mortality of CVD, which is increasing due to the aging of the population and the rising prevalence rates of diabetes and obesity. In this review, we will summarize the different therapeutic peptides developed or used focused on the treatment of myocardial IR injury (MIRI). Therapeutic peptides will be presented depending on their interacting mechanisms (apoptosis, necroptosis, and inflammation) reported as playing an important role in reperfusion injury following myocardial ischemia. The search and development of therapeutic peptides have become very active, with increasing numbers of candidates entering clinical trials. Their optimization and their potential application in the treatment of patients with AMI will be discussed.

## Introduction

### Epidemiology

According to the World Health Organization (WHO), cardiovascular diseases (CVD) are the number one cause of death worldwide representing 31% of all global deaths and 18.6 million lives per year (1). CVD's burdens are predicted by WHO to stay at the first rank until 2030 due to the aging of the population and the increasing prevalence of diabetes and obesity.

Acute myocardial infarction (AMI) among CVD represents the first cause of mortality worldwide (15.9 million/year). Since more than two decades, myocardial ischemia-reperfusion injury (MIRI) has been investigated resulting in important progress in both the knowledge of the mechanisms underlying cell death and in improved interventional procedures. However, MIRI is still associated with significant mortality and morbidity since 30% of infarcted patients die and 25–50% of survivors develop heart failure (HF) representing huge societal costs.

Furthermore, experts predict that the global burden of cardiovascular disease will grow exponentially over the next few years because of the increased prevalence of diabetes and due to the long-term effects of the current COVID-19 pandemic (2, 3).

### Myocardial Ischemia-Reperfusion Injury

AMI is a consequence of the complete coronary artery occlusion occurring at the site of a plaque rupture, exposing its inner core and thus promoting thrombus formation. Criteria for AMI (types 1, 2, and 3 MI) are based on the presence of a myocardial injury with clinical evidence of acute myocardial ischemia and detection of a rise and/or fall of cardiac troponin (cTn) values, associated with at least one of the following symptoms: myocardial ischemia, new ischemic electrocardiographic (ECG) changes, development of pathological Q wave, new regional wall motion abnormality or detection of a thrombus [see the universal definition (4)].

For all types of MI, rapid restoration of blood flow in the ischemic myocardium leading to myocardial reperfusion, either by thrombolysis or angioplasty, has become the “cornerstones” of treatment for AMI (5, 6). All recommendations agree that reperfusion therapy should be performed in patients within the first 12 h of infarction to limit infarct size (IS), improve survival and prevent post-ischemic HF (7). However, sudden blood flow restoration leads to fatal damage to cardiac cells *via* the activation of various intracellular cascades (8). Since the initial description of this phenomenon by Jennings et al. (9) near 40 years ago, our understanding of the mechanisms of reperfusion injury has increased considerably. The pathogenicity is linked mainly to regulated cell death (RCD) under the control of numerous biochemical and cellular processes such as a burst of reactive oxygen species (ROS), loss of ionic homeostasis, mitochondrial dysfunction, and inflammation (10). Lethal reperfusion injury, additional to ischemic injury, culminates in apoptotic death of cardiac cells that were viable immediately before myocardial reperfusion. Because the adult heart fails to have quantities of endogenous stem cells for cardiac regeneration (11), dead cardiomyocytes are lost forever. At the moment, no pharmacological treatment is available to prevent reperfusion injury (12, 13).

### Modes of Cell Death During MIRI

The mechanisms contributing to IR injury are multifactorial and highly integrated (10). The existence of such mechanisms triggered by reperfusion and leading to deleterious side effects including cell death can explain the persistence of significant mortality despite early patient management and the development of chronic HF in a significant proportion of reperfused AMI (14). Indeed, reperfusion triggers cascades of biochemical and metabolic events that aggravate changes generated during ischemia. Studies in animal models suggest that reperfusion lesions are responsible for 25 to 50% of the final IS (15).

Cells can die from accidental cell death (ACD) (such as necrosis), which is usually triggered by unexpected injury or attack, escaping from any controlled molecular mechanisms (16, 17). Qualitatively, necrosis is the major mechanism of cardiomyocyte death during ischemia as a direct consequence of oxygen deprivation leading to severe injury. In contrast, during reperfusion, cardiomyocytes die from regulated cell death (RCD) mechanisms involving genetically defined effector molecules and precise signaling cascades such as apoptosis and necroptosis (8).

#### Apoptosis

**Figure 1:**
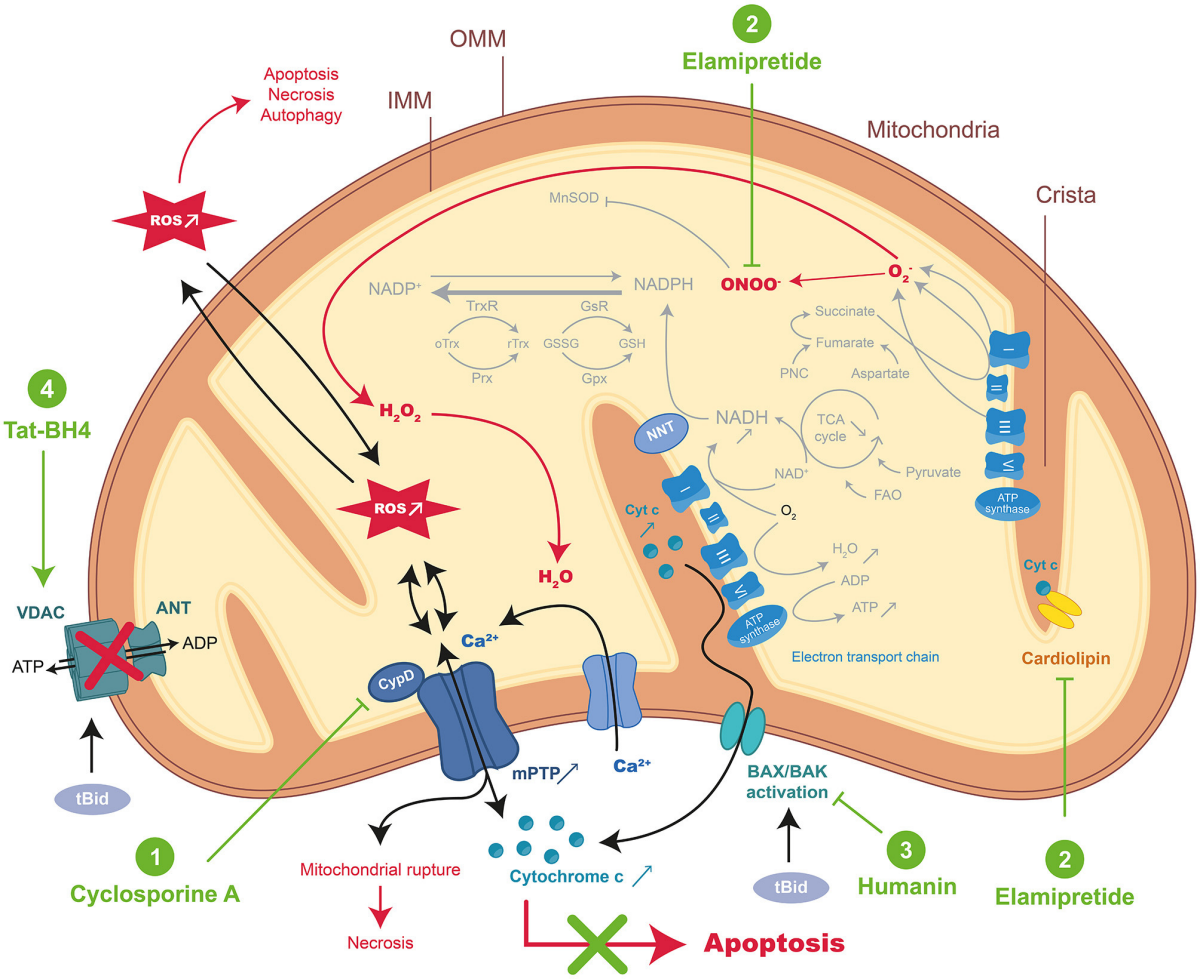
Schematic representation of mitochondrial-dependent apoptosis and mode of action of the therapeutic peptides during MIRI. During acute myocardial ischemia-reperfusion injury (MIRI), reactive oxygen species (ROS) burst and mitochondrial Ca^2+^ overload activate regulated cell death (RCD) resulting in apoptosis or necrosis through the mitochondrial permeability transition pore (mPTP) opening. Excessive ROS induced important changes in normal mitochondrial structure and function resulting in the disorder of mitochondrial metabolic function. Therapeutic peptides reducing intrinsic apoptosis during MIRI were highlighted in green: (1) Cyclosporine A (CsA), (2) Elamipretide, (3) Humanin, and (4) Tat-BH4. CypD, cyclophilin D; NNT, nicotinamide nucleotide transhydrogenase; FAO, fatty acid β-oxidation; Prx, peroxiredoxins; Gpx, glutathione peroxidase; GsR, glutathione reductase; Trx, thioredoxin; TrxR, thioredoxin reductase; GSH, glutathione; GSSG, oxidized glutathione; PNC, purine nucleotide cycle; tBid, truncated form of BH3 Interacting domain Death agonist; BAX, BCL2 associated X Apoptosis regulator; BAK, BCL-2 Antagonist/Killer 1; OMM, outer membrane, IMM, inner membrane.

**Figure 2:**
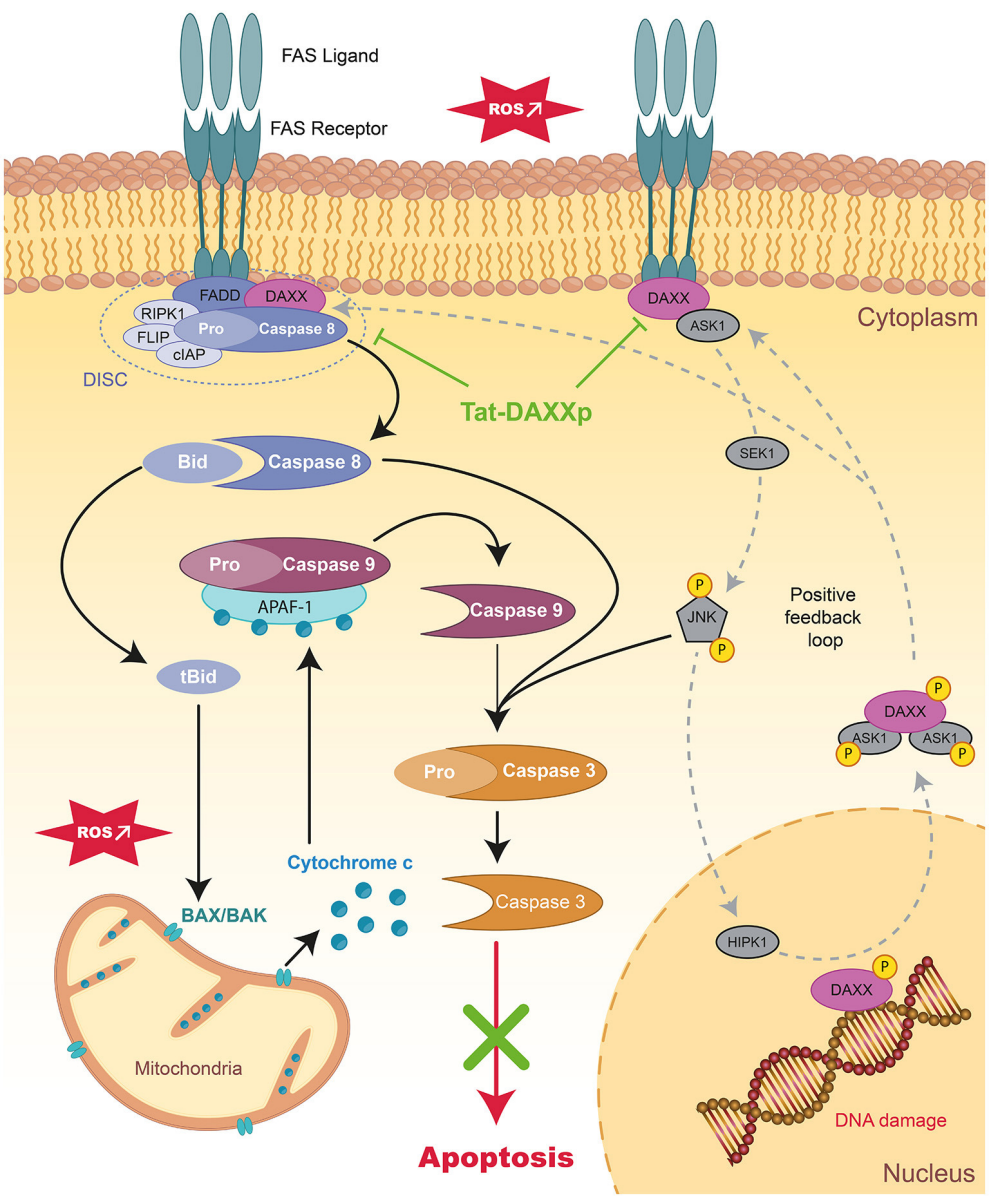
Schematic representation of extrinsic apoptotic pathways and mode of action of therapeutic peptides during MIRI. Schema presenting the signaling apoptotic cascades activated during MIIR involving receptor-dependent pathways in the myocardium. The positive feedback loops regulating DAXX nucleic-cytoplasmic ratio is showed in dark gray. Tat-DAXXp treatment administered at the onset of reperfusion (highlighted in green) can inhibit both the extrinsic and intrinsic pathways. FAS, First Apoptosis Signal; FADD, Fas-Associated protein with Death Domain; DAXX, Death-domain associated protein-6; DISC, death-inducing signaling complex; RIPK1/3, Receptor-interacting serine/threonine-protein kinase 1/3; FLIP, FLICE-inhibitory protein; cIAP, cellular inhibitor of apoptosis proteins 1; ASK1, Apoptosis Signal regulating Kinase 1; JNK, c-Jun N-terminal Kinase; HIPK, homeodomain-interacting protein kinase.

Characterized by cell shrinkage, chromatin condensation, and distinctive blebbing (budding) of the plasma membrane, occurs *via* the intrinsic (Figure 1) or extrinsic (Figure 2) pathways converging into Caspase-activation and DNA fragmentation as a common result (18). Specific DNA fragmentation is mainly detected in the myocardium after reperfusion and not during ischemia, suggesting that its activation is specifically triggered at the onset of reperfusion, depending on the recovery of ATP production (19, 20). This is supported by a major IS reduction observed after the administration of inhibitors of pro-apoptotic mediators at early reperfusion (21, 22). Moreover, the gold standard of cardioprotection, ischemic postconditioning, inhibits apoptosis as does the ischemic preconditioning from which it is derived (23–25). Cardioprotection results from anti-apoptotic strategies (18) such as peptides targeting the First Apoptosis Signal (FAS) death-dependent apoptotic receptor (26, 27), or the mitochondrial permeability transition pore (mPTP) such as Cyclosporine-A (CsA) with however inconsistent preclinical and clinical results (28).

#### Necroptosis

**Figure 3:**
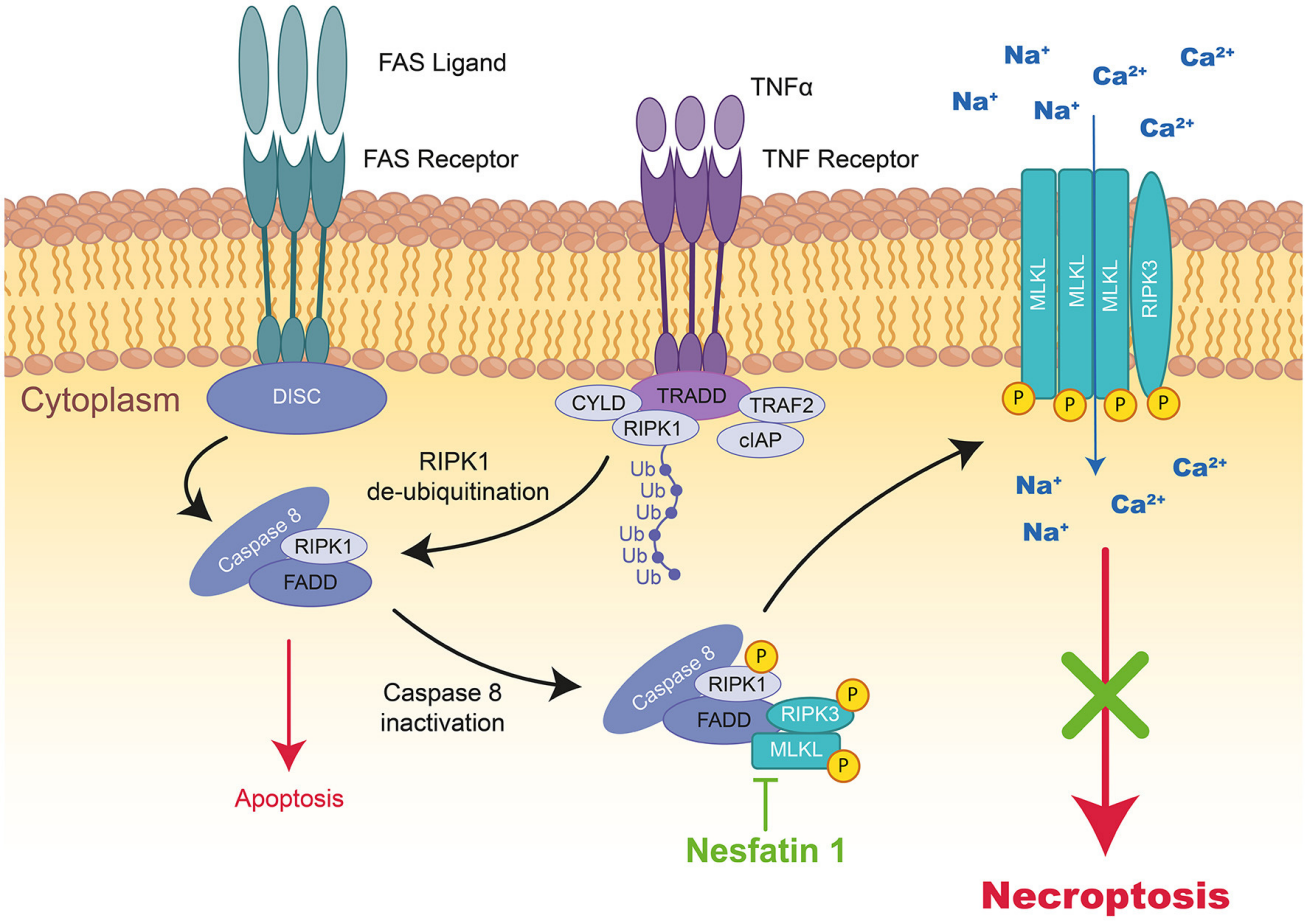
Schematic representation of necroptosis and the mode of action of Nesfatin 1 therapeutic peptide during MIRI. TNFα activates the TNF receptor, which induces the formation of a complex formed by TRADD, TRAF2, RIPK1, CYLD, and cIAP1 at the cytoplasmic membrane. In the absence of cIAP1, RIPK1, FADD, and Caspase-8 form cytosolic DICS complex, Caspase-dependent pathways are activated inducing apoptosis. However, by Caspase-8 inactivation, RIPK1 interacts with RIPk3 and MLKL to form a third complex inducing necroptosis. The kinase of RIPK1 phosphorylates RIPK3 and MLKL resulting in their translocation to the plasma membrane, where the complex mediates membrane permeabilization. The therapeutic peptide Nesfatin-1 (highlighted in green) can reduce RIPK1, RIPK3, and MLKL expression and therefore necroptosis. TNF, tumor necrosis factor; TRADD, tumor necrosis factor receptor type 1-associated death domain; TRAF2, TNF receptor-associated factor 2; RIPK1/3, receptor-interacting serine/threonine-protein kinase 1; CYLD, lysine 63 deubiquitinase; cIAP1, cellular inhibitor of apoptosis protein 1; MLKL, mixed lineage kinase domain like pseudo kinase.

In cardiac pathology, necroptosis has been identified as a lytic form of RCD leading to the release of proinflammatory intracellular molecules (29, 30). Even if necroptosis is morphologically similar to necrosis, this pathway could also depend on Caspase-8 activity and therefor, be pharmacological modulable (i.e., inhibited by necrostatin-1). Necroptosis is triggered by oxidative stress or TNFα (Tumor Necrosis Factor), FasL (FAS Ligand), and TRAIL (TNF-Related Apoptosis-Inducing Ligand) cytokines activating death receptors (Figure 3) (31). Necroptosis is triggered only if Caspase-8, responsible for the cleavage of RIPK1 (Receptor-Interacting serine/threonine-Protein Kinase 1), is inhibited. Phosphorylated RIPK1 and RIPK3 together with MLKL (Mixed Lineage Kinase Domain Like Pseudokinase) form the necrosome leading to phosphorylation and oligomerization of MLKL, which translocates to the plasma membrane to induce membrane rupture (32).

#### Autophagy-Dependent Cell Death

Autophagy is an evolutionary process to maintain cell homeostasis based on the degradation of intracellular materials and components within the lysosomal compartment of eukaryotic cells (17). Because of the elimination of misfolded/dysfunctional proteins or organelles, autophagy was believed to be a cytoprotective catabolic mechanism of substrate recycling for ATP generation and cell survival. Autophagy is activated by ATP-depletion and subsequent AMPK (AMP-dependent Protein Kinase) activation, calcium overload, and ROS, which are found during prolonged ischemia, IR, and HF (33, 34). A high level of Beclin-1 is critical for early autophagosome formation and its activity can be reduced by BCL-2 (B-cell lymphoma-2) or BCL-XL (B-cell lymphoma extra-large). Beclin-1 is cleaved by Caspases, showing the existence of a crosstalk between autophagy and apoptosis (35). More recently, autosis was described as a new form of autophagy responsible for continuous cardiomyocyte death in the late phase of reperfusion although cell death processes should be completed within 2 h of reperfusion (36, 37).

#### Inflammation

**Figure 4:**
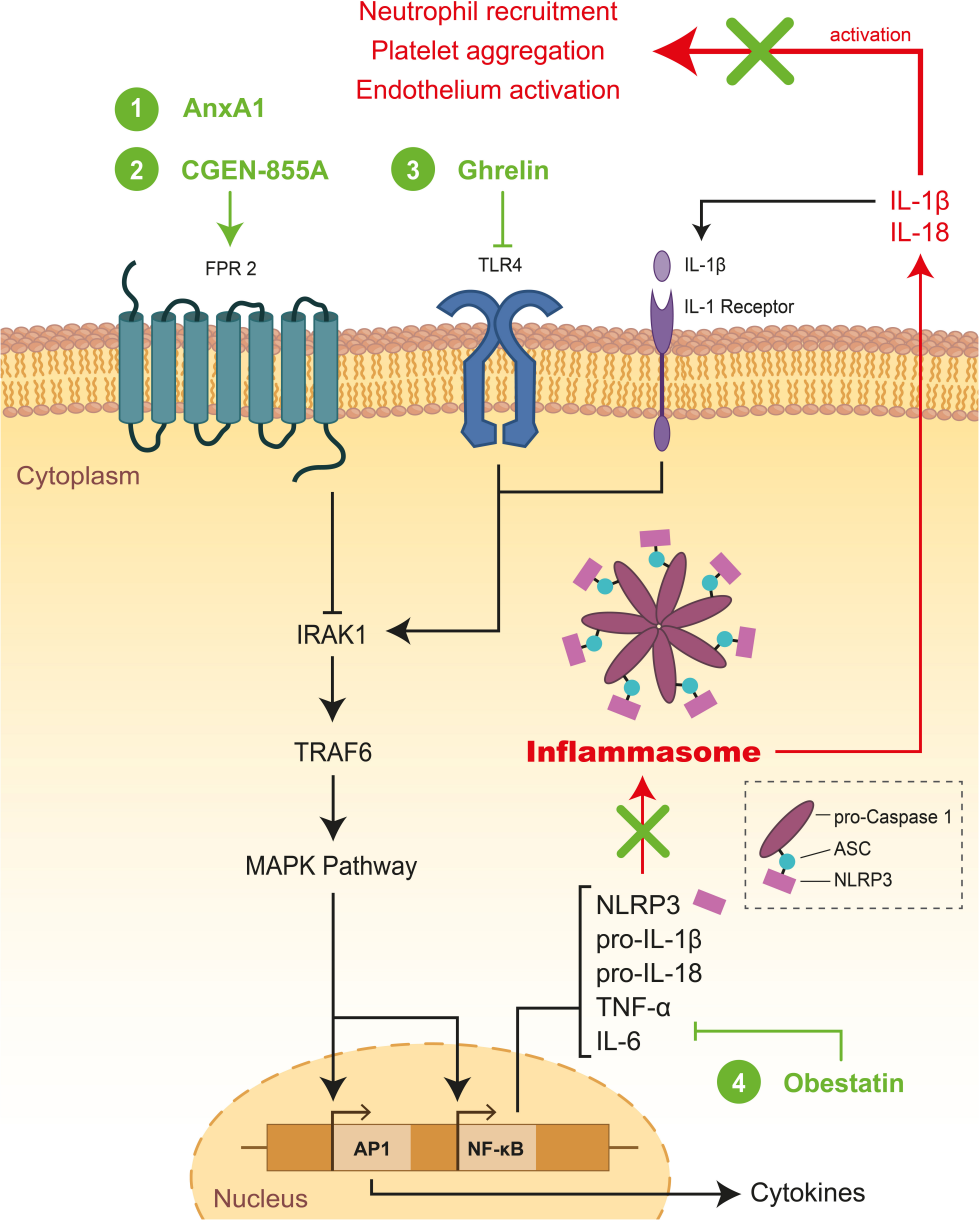
Schematic representation of inflammation and mode of action of therapeutic peptides during MIRI. FPR, TLR4, or IL1 receptors activate the MAPK pathway through IRAK1 and TRAF6. Afterward, the MAPK pathway activates the expression of NLRP3 which formed the inflammasome together with pro Caspase-1 and ASC (Apoptosis-associated speck like protein containing a Caspase recruitment domain) contributing to the strong but short inflammatory burst. Secretion of mature forms of IL-18 and IL-1β activate neutrophil recruitment, platelet aggregation, and endothelium activation. Therapeutic peptides reducing inflammation during MIRI were highlighted in green: (1) AnxA1, (2) CGEN-855A, (3) Ghrelin, and (4) Obestatin. FPR, Formyl peptide receptor; TLR4, Toll-Like Receptor 4; IL1, Interleukin 1; IRAK1, Interleukin 1 Receptor Associated Kinase 1; TRAF6, TNF receptor-associated factor 6; NLRP3, NOD-, LRR- and pyrin domain-containing protein 3.

Necrotic cardiomyocytes in the infarcted area provide the main stimulus for post-infarction inflammatory response through the release of DAMPs (Damage-Associated Molecular Patterns) in concert with complement cascade and ROS activation, mobilizing the resident immune cells of the heart at the onset of AMI. Neutrophil infiltration, innate immunity activation as well as cell-mediated damage are pathological mechanisms of inflammation-related IR injury observed after MI as exemplified through experimental and clinical studies (38). In the context of AMI, the pro-inflammatory response, rapidly orchestrated during ischemia to remove the necrotic cells and repair the infarcted myocardium, is exacerbated following reperfusion leading to cardiomyocyte death and MIRI within 6 h and 24 h post-reperfusion. TLR2, TLR4, TLR9 (Toll-Like Receptors) and NLRP3 in the NLRP3-ASC-Caspase-1 inflammasome contribute to this strong but short inflammatory burst following reperfusion through IL-1 (Interleukin-1), IL-6, and active Caspase-1 mediators *via* the NF-κB pathway (Figure 4) (39). Inflammation is deeply involved in the pathophysiology of MIRI but also in fibrosis formation and in post-infarct remodeling leading to HF (40). Because inflammation contributes to IS and cardiac remodeling, it is a major predictor of adverse events after AMI (41, 42).

### Therapeutic Peptides as a Novel Approach for Treating MIRI?

Important chemical development of solid-phase peptide synthesis allowed the rise of not only small but also larger synthetic peptides. In this review, peptides will be defined as molecules containing no more than 30 amino acids, to discriminate them from proteins or antibodies, which constitute a large field within the pharmaceutical industry. Peptides are attractive therapeutic molecules based on their favorable pharmacokinetic profile, good solubility, low toxicity/mitogenicity, and furthermore due to the unlimited possibility of introducing modifications to improve their stability/binding affinity (43). It is then not surprising that therapeutic approaches using peptides have become an emerging market in the pharmaceutical industry over the past decades and that today more than 60 peptide drugs are Food Drug Administration approved and that many more are studied in clinical and preclinical trials (44). The global peptide therapeutics market accounted for $28.15 billion in 2019 and is expected to reach $66.76 billion by 2027 growing at a CAGR of 11.4% during the forecast period (45).

As a consequence, a large variety of bioactive peptides that target processes of apoptosis, necroptosis, inflammation, and autophagy in MIRI have been identified and characterized (46). The next chapter will be focused on therapeutic peptides administrated at the onset of reperfusion (pharmacological post conditioning) which is the only relevant clinical application since pharmacological preconditioning is incompatible with AMI therapy. Furthermore, we have mainly focused on those validated in *ex vivo* and *in vivo* IR animal models.

## Therapeutic Peptides Reducing Apoptosis To Treat MIRI

Apoptotic mechanisms are dependent on ATP production, which means that this mechanism is not activated in ischemic conditions but is specific to the reperfusion phase (19). Indeed, apoptotic cascades pre-activate during ischemia are fully executed during reperfusion (DNA fragmentation) (47). Accordingly, numerous *in vivo* studies have reported positive results for specific anti-apoptotic interventions as cardioprotective strategies against MIRI (22, 23, 26, 48–50) because apoptosis, through the intrinsic (mitochondrial) (17) and extrinsic (death receptor-dependent) (17, 26) pathways, is a reversible process and can be manipulated to allow cardiomyocyte survival during AMI. All therapeutic peptides described below are summarized in Table 1 including the used experimental conditions (doses, administration modes, animal models, and potent effects), and those evaluated in clinical trials are listed in Table 2.

**Table 1:** Therapeutic peptides used in different IR animal models mentioned in this review.

**Peptides**	**Other names**	**Experimental models**	**Administration**	**Species**	**Mechanisms**	**References**
**MITOCHONDRIA-DEPENDENT APOPTOSIS**						
Cyclosporine A	Ciclosporine A, CsA, CycloMulsion, Sandimmune	*in vivo* 30 min I/3 h R	10 mg/kg, i.v.−5 min before R	Rabbit	Reduced IS	(51)
		*in vivo* 30 min I/2 h R	10 mg/kg, i.v.—at the onset of R	Mouse	Reduced IS	(52)
		*in vivo* 25 min I/2 h R	5 mg/kg, i.v.−5 min before R	Rat	Reduced IS	(53)
		*in vivo* 90 min H/2 h R	5 mg/kg, i.v.−5 min before R	Pig	Reduced IS	(54)
		*in vivo* 40 min I/3 h R	10 mg/kg, i.v.−5 min before R	Pig	No reduction in IS	(55)
		*in vivo* 45 min I/2 h R	10 mg/kg, i.v.−3 min before R	Pig	No reduction in IS	(56)
		*in vivo* 40 min I/4 h R	2.5 mg/kg, i.v.−7 min before R	Pig	No reduction in IS	(57)
		*in vivo* 60 min I/3 h R	10 mg/kg, i.v.−15-10 min before R	Pig	Reduced IS and microvesicular damage, better LV function	(58)
Elamipretide	SS-31, MTP-131, Bendavia	*ex vivo* 30 min global I/3 h R	1 μmol/L—during the whole R	Guinea pig	Improved contractile force, increased heart beating rates	(59)
		*ex vivo* 20 min global I/2 h R	post-ischemic administration 1 nM during R	Guinea pig	Reduced IS	(60)
		*in vivo* 30 min I/180 min R	at the onset of R−0.10 mg/kg/h i.v. during 20 min then 0.05 mg/kg h i.v. during 160 min	Rabbit	No significant effect on IS	
		*in vivo* 60 min I/180 min R	0.05 mg/kg/h i.v.—during the 30 min before R	Sheep	Reduced IS, protection against the no-reflow phenomenon	
		*ex vivo* 20 min global I/120 min R	10 μmol/L i.v.—at the onset of R	Rat	Improve mitochondria function by aggregating cardiolipin	(61)
Humanin	S14G-humanin	*in vivo* 45 min I/24 h R	2 mg/kg i.c.—at the onset of R	Mouse	Reduced IS and increased EF	(62)
		*in vivo* 30 min I/2 h R	252 μg/kg i.v.−15 min before R	Rat	Reduced IS, arrhythmia, and cardiac mitochondrial dysfunction	(63)
		*in vivo* 60 min I/48 h R	2 mg/kg i.v.−10 min before R	Pig	Reduced IS	(64)
Tat-BH4	/	*in vivo* 40 min I/24 h R	1 mg/kg i.v.—at the onset of R	Mouse	Reduced IS and apoptosis	(65)
Tat-V1-Cal	/	*in vivo* 30 min I/2 h R	1 mg/kg i.v.−5 min before R	Rat	Reduced IS	(66)
AID-Tat	/	*in vivo* 30 min I/12 weeks R	10 μmol/L i.c.—at the onset of R	Rat	Reduced IS and supported contractility	(67)
**RECEPTOR-DEPENDENT APOPTOSIS**						
Tat-DAXXp	TD	*in vivo* 40 min I/24 h R	1 mg/kg i.v.—at the onset of R	Mouse	Reduced IS and apoptosis	(26)
		*in vivo* 40 min I/6 months R	1 mg/kg i.v.—at the onset of R	Mouse	Reduced fibrosis, increase EF	(27)
Tat-FADDp	TF	*in vivo* 40 min I/24 h R	1 mg/kg i.v.—at the onset of R	Mouse	Reduced IS and apoptosis	(68)
**INFLAMMATION**						
ANP	ANP1-28	*ex vivo* 15 min I/15 min R	0.1 μmol/L—at the onset of R	Rat	Better cardiac and corona flow recovery	(69)
	Carperitide	*in vivo* 60 min I/6 h R	0.2 μg/kg/min i.v.−15 min after I till the end of R	Dog	Reduced IS, increased blood flow, decreased left ventricular systolic pressure, and end-diastolic pressure	(70)
		*in vivo* 90 min I/6 h R	0.1 μg/kg/min i.v.−10 min before I till 1 h of R	Dog	Reduced IS	(71)
	Urodilatin (URO)	*ex vivo* 40 min I/1 h R	0.05 μmol/L—first 15 min of R	Rat	Higher LV pressure	(72)
		*in vivo* 47 min I/2 h R	10 ng/kg/min i.v.—during the first 25 min of R	Pig	Reduced IS	
BNP	/	*ex vivo* 30 min I/90 min R	10 nmol/L−5 min before R till 15 min of R	Rat	Reduced IS	(73)
	/	*in vivo* 30 min I/4 h R	0.03μg/kg min i.v.−15 min before R till the end of R	Rat	Reduced IS, decreased LDH, and CK levels	(74)
	RhBPN	*in vivo* 40 min I/24 h R	0.035 mg i.p.—after IR one injection/d for 3 d	Mice	Reduced IS and CD4+T cell proliferation	(75)
DNP	Lebetin 2 (L2)	*ex vivo* 30 min I/90 min R	200 nmol/L−5 min before R till 15 min of R	Rat	Reduced IS	(73)
		*in vivo* 30 min I/2 h R	100 ng/g i.p.−5 min before R	Mouse	Reduced IS	(76)
		*in vivo* 35min I/2 d or 14 d R	25 ng/g i.p.−5 min before R	Rat	Reduced IS, collagen content, and enhanced M2-like macrophages	
CNP	/	*ex vivo* 25 min I/2 h R	30 nmol/L—during the first 30 min of R	Rat	Reduced IS and coronary perfusion pressure (CPP)	(77)
VNP	Vasonatrin	*in vivo* 30 min I/4 h or 6 h R	100 μg/kg, i.v.−10 min before R	Rat	Reduced IS, Reduced LV systolic and end-diastolic pressure	(78)
Ac2-26	N-terminus of Annexin-1	*in vivo* 25 min I/2 h R	1 mg/kg i.v.—at the onset of R	Rat	Reduced IS, Myeloperoxidase (MPO) activity, and IL-1b levels	(79)
		*in vivo* 40 min I/24 h R	1 mg/kg i.v.—at the onset of R	Mouse	Reduced IS, cTnI (24 hR), inflammation (48 h R), fibrosis, and apoptosis (7-days R)	(80)
AnxA1_2−50_		*in vivo* 25 min I/2 h R	5 μg/mouse i.v.—at the onset of R	Mouse	Reduced IS and plasma levels of cTnI, CCL5, ILβ	(81)
CR-AnxA1_2−50_						
CR-AnxA1_2−48_		*in vivo* 25 min I/2 h R	5 μg/mouse i.v.—at the onset of R	Mouse	Reduced IS and plasma CCL5 concentration	(82)
CGEN-855A	/	*in vivo* 25 min I/2 h R	2 mg/kg i.v.—immediately after R	Mouse	Reduced IS, cTnI and PMN	(83)
		*in vivo* 30 min I/3 h R	2 mg/kg i.v.−5 min before R	Rat		
DS-IkL	/	*in vivo* 45 min I/24 h R	30 μM 100 μL i.v.—immediately after R	Mouse	Reduced IS and cTnI concentration	(84)
Ghrelin	/	*ex vivo* 30 min global I/30 min R	10,000 pM—during R	Rat	Increased coronary flow, heart rate, left ventricular systolic pressure and left ventricular end-diastolic pressure	(85)
		*in vivo* 30 min I/24 h R	8 nmol/kg i.v.—at the onset of R	Rat	Reduced IS, inhibition of the TLR4, NLRP3, and Caspase-1 expression	(86)
Obestatin	/	*in vivo* 30 min I/24 h R	50 nM/kg—LV injection at the R	Rat	Reduced IS	(87)
		*ex vivo* 30 min I/2 h R	75 nM—during the first 20 min of R	Rat	Reduced IS	(88)
**NECROPTOSIS**						
Nesfatin-1	/	*in vivo* 30 min I/24 h R	20 μg/kg i.p.—prior R	Rat	Reduced IS and EF, Reduced Beclin-1 and Caspase-3 expression	(89)
		*in vivo* 30 min I/24 h R	20 μg/kg i.p.—prior R	Rat	Reduced expression of RIPK1, RIPK3, MLKL, ROCK1, and ROCK2 proteins	(90)
		*in vivo* 30 min I/2 h R	100 pmol/L—during the first 20 min of R	Rat	Reduced IS, ERK1/2 activation	(91)
**AUTOPHAGY**						
HBSP	Helix B surface peptide	*in vivo* 45 min I/2 h R	90 μg/kg, i.p.−5 min before R	Mouse	Reduced IS, decreased cardiomyocyte apoptosis	(92)
**OTHERS**						
GLP-1	Glucagon-like peptide 1; [GLP-1(7–36)amide]; Exenatide	*ex vivo* 45 min global I/2 h R	0.3 nM—at the onset of R	Rat	Improve LV pressure, no effect on IS	(93)
		*in vivo* 30 min I/2 h R	4.8 pmol/kg/min—perfusion during the procedure	Rat	Reduced IS	(94)
Apelin-13/-36	/	*ex vivo* 35 min global I/35 min R	1,000 nM Apelin-13/1,000 nM Apelin-36—at the onset of R	Rat	Reduced IS (Apelin-13 = 40%/Apelin-36 = 26%)	(14)
		*in vivo* 30 min I/120 min R	0.1 mg/kg Apelin-13/0.27 mg/kg Apelin-36, i.v.—at the onset of R	Mouse	Reduced IS (Apelin 13 = 43%/Apelin 36 = 33%)	
Apelin-13	/	*in vivo* 45 min regional I/24 h R	0.1 μg/kg—at 5 min after R	Mouse	Reduced IS, decreased apoptosis	(95)
Elabela	Apela; Toddler	*in vivo* 30 min regional I/4 h R	0.7 mg/kg, i.v.—at 5 min of R	Rat	Decreased apoptosis, fibrosis, and oxidative stress	(96)

*H, hypoxia; I, ischemia; R, reperfusion; i.p, intra peritoneal injection; i.v, intra venous injection; i.c., intra coronary injection; IS, infarct size; LV, left ventricle*.

**Table 2:** Therapeutic peptides used in clinical trials mentioned in this review.

**Peptides**	**Clinical trial**	**Administration**	**Nb of patients**	**Results**	**References**
Elamipretide	EMBRACE	0.05 mg/kg/h, between 60–15 min before PCI and for 1 h following reperfusion	297	**no IS reduction (CK-MB** **quantification)**	(97)
Cyclosporine A	/	2.5 mg/kg, catheter in the antecubital vein, <10 min before direct stenting	57	IS reduction	(98)
	CIRCUS	2.5 mg/kg, i.v., 12 h within symptom onset	970	**not better than placebo**	(99)
	CYCLE	2.5 mg/kg, i.v., 6 h within symptom onset	410	**no effect on ST-segment resolution or** **hs-cTnT, no improved clinical** **outcomes or LV remodeling up to 6** **months**	(100)
	CYRUS	2.5 mg/kg, i.v., asap after the onset of ACLS	6,758	**do not prevent early multiple organ** **failure**	(101)
Carperitine	/	0.085 μg/kg/min i.v. for 65 h	3,777	better outcome	(102)
	J-WIND	0.025 μg/kg/min i.v. for 3 days	1,216	Reduced IS, increased LV EF, decreased reperfusion injury, **severe hypotension**	(103)
	AVCMA	0.0125–0.025 mg/kg i.v.	111	higher plasma BNP level, reduced blood pressure, **hypotension**	(104)
Nesiritide	/	0.01–0.03 μg/kg	862	**Increased risk of death after treatment**	(105)
	/	≤ 0.03 g/kg/min i.v.	1,269	**Increased renal disfunction**	(106)
Exenatide	/	25 μg/250 mL i.v. 15 min before intervention and maintained 6 h	172	Reduced IS, larger salvage index	(107)
	/	20 μg during PCI and 10 μg twice daily during 48 h	58	Reduced IS, improved LV function	(108)
	/	10 μg/h 30 min and 0.84 μg/h 72 h	191	**No benefit**	(109)
	COMBAT-MI	18 μg/180 mL i.v. 15 min before intervention and maintained 6 h combined with RIC procedure	222	**No benefit**	(110)

*i.v, intra venous injection; IS, infarct size; LV, left ventricle; PCI, percutaneous coronary intervention; negative outcomes of clinical trials were highlighted in bold*.

### Therapeutic Peptides Acting on the Intrinsic Apoptotic Pathway

The intrinsic or mitochondrial pathway is activated upon intracellular stress such as calcium overload or damaged DNA and is characterized by the irreversible permeabilization of the mitochondrial outer membrane under the control of proteins with BCL-2 homology domain and a transmembrane segment (111). The formation of pores in the outer membrane by oligomerization of BAX (BCL2 associated X Apoptosis regulator) and BAK (BCL-2 Antagonist/Killer 1) is regulated by activating BH3-only proteins such as Bid (BH3 Interacting-Domain death agonist) and by sensitizers such as BAD (BCL2 associated Agonist of cell Death) that sequester the anti-apoptotic proteins (BCL-XL) previously bound to direct activators.

Moreover, in addition to its activation by Caspase-8 or granzyme B, Bid is engaged in response to death receptor stimulation, allowing crosstalk between the intrinsic and extrinsic pathways (112). The permeabilization of the outer membrane leads to the release of pro-apoptotic factors into the cytoplasm, endonuclease G that will cleave DNA (Caspase-independent mechanism) or SMAC (Second Mitochondrial Activator of Caspases) and cytochrome C (CytC) inducing *in fine* DNA fragmentation and cell apoptosis (113). Mitochondrial respiratory chain with reduced CytC leads to mitochondrial ATP synthesis dysfunctions and to the dissipation of the mitochondrial transmembrane potential (Δψm), which in turn triggers the opening of the mPTP (Figure 1) (114). mPTP is a protein complex whose molecular identity remains not fully elucidated. Several proteins have been reported to be part of this complex such as VDAC (Voltage-Dependent Anion Channel) located in the outer membrane and ANT (Adenine Nucleotide Translocator) spanning the inner membranes, whereas others are described as protein regulators, such as the mitochondrial matrix chaperone cyclophilin D (CypD) (114, 115). The mPTP formation and opening occur at the onset of reperfusion (116) upon (i) oxidative stress when the respiratory chain is suddenly exposed to oxygen, (ii) Ca^2+^ ion accumulation due to rapid mitochondrial membrane potential restoration, and (iii) neutralization of acidic pH as H^+^ ions compete with Ca^2+^ ions that bind to the mPTP trigger site (117). Furthermore, mPTP allows the passage of small molecules (<1.5 kDa) into the mitochondrial matrix (118) and will contribute to the permeabilization of the mitochondrial inner membrane.

mPTP opening has been proposed as the key driver of MIRI because the concentration of the endogenous potentiators of the mPTP (e.g., calcium and ROS) increased during this phenomenon whereas inhibitors (e.g., Cyclosporine A) reduced IS (119). However, a major limitation of mPTP inhibiting is the lack of knowledge on mPTP-forming proteins and how they are activated by calcium and ROS.

#### Cyclosporine A

Cyclosporine A (CsA) is a natural cyclic 11-mer peptide, isolated from fungus *Tolypocladium inflatum*, widely used to down-regulate immune system activity and therefore the risk of organ rejection after allogeneic organ transplant. CsA is able to block mitochondrial calcium efflux in isolated mitochondria and to inhibit the Ca^2+^-dependent mPTP opening in the inner membrane of heart mitochondria (120, 121). CsA (122) and the analogs NIM811 (123) and Debio-025 (124) bind mitochondrial CypD, preventing the mPTP pore opening, and have been described as promising drugs for the cardioprotection against MIRI (Figure 1) (119). Indeed, CsA has provided encouraging results in many animal IR models (51–58) (for details see Table 1) and also in a proof-of-concept study in patients (98). More recently, large-scale clinical trials were performed to evaluate CsA long-term cardioprotective effects (CIRCUS, CYCLE, CYRUS) (99–101) (Table 2). Unfortunately, published results have shown no evidence of long-term protection, reduced mortality, or prevention of early multiple organ failure. The controversial findings obtained in clinical studies were attributed to the differences in CsA formulations since it is a class II compound with extremely low aqueous solubility (6.6 g/mL) and high lipophilicity (Log P = 3). However, even if both formulations have been described to have similar pharmacokinetics (125), Sandimmune used in CYCLE and CYRUS trials did not show the expected efficacy previously reported in the proof-of-concept trial by Piot et al. (98, 100, 101). The same negative results were obtained using CicloMulsion®, a lipid emulsion of CsA, in the large-scale CIRCUS clinical trial (99).

More recently, new formulations using PLGA-, squalene- or lipid-based nanoparticles were developed to increase cell permeability of the therapeutic CsA but no preclinical study was yet performed (126–128). Also, combined administration of polymeric nanoparticles encapsulating CsA or pitavastatin (organic compound) targeting mPTP opening and monocyte-mediated inflammation, respectively, has been reported to be more efficient than a single administration of encapsulated CsA (129) even if CsA impacts *per se* the immune response after myocardial IR (130).

#### Elamipretide

The small cell-permeable Szeto-Schiller peptide (also known as SS-31, MTP-131, Bendavia, or Elamipretide) was developed for targeted delivery of antioxidants to the inner mitochondrial membrane (131). This 4-mer SS peptide can scavenge hydrogen peroxide or peroxynitrite and inhibit lipid peroxidation through its structural motif alternating aromatic residues and basic amino acids (Figure 1). Therefore, this SS-31 peptide provides significant protection against MIRI as shown by higher contractile force levels as well as increased heart beating rates, and prevents myocardial stunning when administered upon reperfusion in the *ex vivo* guinea pig heart (59). As a mechanism of action, SS-31 could interact with phospholipids (i.e., cardiolipin) on the inner mitochondrial membrane maximizing membrane shape to improve the electron transport chain function and minimize the production of mitochondrial-derived ROS (Figure 1).

Later on, Kloner and co-workers demonstrated that Elamipretide reduced myocardial IS in different IR models (60). For example, post-ischemic Elamipretide administration decreases IS in an *ex vivo* guinea pig IR model in the same way as in the *in vivo* sheep IR model where it was infused during the last 30 min of ischemia. Surprisingly, the authors could not show any cardioprotection in an *in vivo* rabbit IR model. More recently, Allen and colleagues demonstrated that Elamipretide (10 μM) administrated at the onset of reperfusion in an *ex vivo* rat IR model can improve mitochondrial function by aggregating cardiolipin (61).

Based on these promising experimental data, a multicenter study (EMBRACE STEMI) was performed to evaluate Elamipretide as an adjunct therapy to percutaneous coronary intervention for STEMI. The drug injected to patients before reperfusion was safe and well-tolerated but not associated with a decreased IS as assessed by creatine kinase-myocardial band (CK-MB) quantification (97).

#### Humanin

Humanin (HN) is a mitochondrial-derived polypeptide (24-mer) encoded by mtDNA that regulates mitochondrial functions under stress conditions and protects cells against various situations such as diabetes mellitus, cardiovascular and neurodegenerative diseases mainly through anti-apoptotic effects leading to sequestration of BAX and Bid (132) (Figure 1). Muzumdar and colleagues have shown a significant reduction in IS after an intracardiac administration of HNG (S14G-humanin with a point mutation) at the onset of reperfusion in mice subjected to MIRI. HNG cardioprotection was associated with a significant increase in AMPK and endothelial nitric oxide synthase phosphorylation as well as to attenuation of BAX and BCL-2 levels (62).

Later, Thummasorn and co-workers have demonstrated on rats subjected to MIRI that administration of HNG 15 min before reperfusion decreased IS and arrhythmia (63). Sharp and colleagues confirmed these results in a large animal model of MIRI but these effects were abrogated when ischemic time duration was prolonged from 60 to 75 min (64). Thus, although HNG cardioprotection translates beyond different animal models, further clinical studies are needed to validate HNG therapy for a clinical application.

#### Tat-BH4

Mitochondrial dysfunction and permeability mPTP opening are regulated in part by the voltage-dependent anion channel of the outer mitochondrial membrane (VDAC), which is itself controlled by pro- and anti-apoptotic BCL-2 family members (Figure 1) (133). Based on this fact, Roberta Gottlieb's group designed a peptide corresponding to residues 4–23 of BCL-XL protein conjugated to the protein transduction domain of HIV TAT (TAT-BH4) to develop a cardioprotective therapeutic strategy (134). Indeed, TAT-BH4 preconditioning attenuated CK release and reduced IS in IR rat hearts (15 min before I), demonstrating the role of mitochondria and pro-apoptotic BCL-2 proteins in the process of cell death.

To develop a more physiological-relevant therapeutic application, our group has analyzed the effects of the BH4 peptide injected intravenously at the onset of reperfusion in an *in vivo* murine MIRI model. Among the four formulations of BH4 with various cell-penetrating peptides [CPP: Tat, (RXR)4, Bpep and Pip2b] tested, we observed a decrease of ~47% in IS and ~60% in apoptosis *in vivo* either with Tat-BH4 or Pip2b-BH4 when administered intravenously 5 min before reperfusion (65).

#### Other Therapeutic Peptides Inhibiting the Mitochondrial Pathway

##### V1-cal

Hurt and co-workers have determined that TRPV1 (Transient Receptor Potential Vanilloid 1), a non-selective calcium (ion) channel activated in cellular pain insults including hypoxia, regulates mitochondrial membrane potential and MIRI (66). By using an 11-mer peptide decoy V1-cal coupled to the Tat CPP (135), the authors revealed a substantial reduction in IR injury by inhibiting the inducible calcineurin-TRPV1 interaction in an *in vivo* MIRI rat model.

##### AID-Tat

Viola and colleagues have tested an 18-mer peptide directed against the alpha-interacting domain (AID) of the alpha 1c subunit of L-type calcium channel vectorized by Tat (AID-Tat peptide), which has been shown to attenuate the increase in mitochondrial membrane potential and metabolic activity after activation of the channel (136). Later, they showed that AID-Tat peptide was able to reduce IS in rat hearts exposed to IR injury *ex vivo* when administered immediately after reperfusion (67). AID-Tat peptide was reported to significantly decrease IS and improve cardiac contractility up to 12 weeks post-MI in rats *in vivo* as a result of a decrease in metabolic demand during reperfusion.

### Therapeutic Peptides Inhibiting the Extrinsic Apoptotic Pathway

For several years, we and several other laboratories have confirmed that the death receptor-dependent apoptotic (or extrinsic) pathway is activated during IR injury (137, 138) since elevated FasL levels were found in the blood of AMI patients activating the FAS death-receptor pathway and triggers the downstream apoptotic signaling pathway (20). DAXX (Death-domain associated protein-6) protein acting as downstream FAS receptor adapter appears to play a key role in IR injury in various organs including the heart (50, 139, 140). The different roles of the DAXX protein depend on its subcellular localization: (i) anti-apoptotic in the nucleus and (ii) pro-apoptotic in the cytosol upon the Apoptosis Signal regulating Kinase 1 (ASK1)-shuttling triggered by various stimuli such as oxidative or ischemic stresses (141).

Therefore, we have focused on the development of a therapeutic peptide targeting the FAS:DAXX interaction as a new treatment against MIRI. We designed a 16-mer interfering peptide DAXXp by SPOT synthesis vectorized with the Tat CPP resulting in the conjugated ***Tat-DAXXp*** peptide (26). Our study showed that Tat-DAXXp (1 mg/kg, i.v. 5 min before reperfusion) treatment resulted in 48%-decreased IS in a murine IR model when assessed after 24 h reperfusion. Tat-DAXXp cardioprotection was achieved through the inhibition of both extrinsic and intrinsic apoptotic pathways (Figures 1, 2) and the activation of pro-survival cascades. More impressively, Tat-DAXXp showed the same cardioprotection in a 6-month follow-up study using the same drug/ischemic protocol (27). In brief, Tat-DAXXp treatment decreased by 70% plasma cTnI concentration and mortality assessed at 24 h post-MI, and furthermore, increased ejection fraction (24%) compared to the non-treated control group during the 6-month follow-up. At the end of the protocol, histological analysis revealed a 54%-decreased left ventricular fibrosis content compared to non-treated mice. Remarkably, Tat-DAXXp was still efficient after a 30-min delayed administration after reperfusion showing a wide therapeutic time window of cardioprotection.

In conclusion, targeting the extrinsic pathway with Tat-DAXXp peptide at the onset of reperfusion revealed potent upstream cardioprotection in a murine model of MIRI validating this peptide as a promising candidate for therapeutic application since it promotes both cell survival and improves cardiac contractile function.

## Therapeutic Peptides Reducing Inflammation TO Treat MIRI

### Formyl Peptide Receptor Binding Peptides

Annexin A1 (AnxA1) is a 37 kDa glucocorticoid-regulated protein known to regulate the termination of inflammation and to have a therapeutic potential in IR injury (142). Its N-terminal peptide Ac2-26 was shown to bind the formyl peptide receptor (FPR) family which inhibits neutrophil adhesion, migration, and infiltration (81, 143, 144). In 2001, the group of Perretti has reported that ***Ac2-26*** (1 mg/kg, i.v.) administrated at the onset of reperfusion or during 60 min, revealed significant cardioprotection associated with lower myeloperoxidase activity and IL-1β levels a rat IR model (79). More recently, Qin and co-workers have confirmed a significant reduction in inflammation (48 h post R) associated with decreased IS, fibrosis, and apoptosis (7-days post R) in a murine IR model (80).

In parallel, Perretti and colleagues worked on several longer and modified Annexin A1 peptides called AnxA1_2−50_, CR-AnxA1_2−50_ (81), and CR-AnxA1_2−48_ (82) displaying cardioprotective properties leading to reduced IS and decreased systemic concentration of the Chemokine C-C motif ligand 5 in a murine IR model. Mechanistically, the three peptides act as new Lipoxin A4 receptor agonists impacting phagocyte responses resulting in protective actions.

Knowing that also agonists of formyl-peptide receptor-like 1 displayed cardioprotective effects in IR models, Hecht and co-workers have developed the ***CGEN-855A*** peptide (83) providing cardioprotection in both murine and rat IR models and displaying anti-inflammatory activity as revealed by polymorphonuclear neutrophil inhibition.

Based on the selectin binding sequence of EC-SEAL (145), Dehghani and colleagues have created the 7-mer ***DS-IkL peptide*** using the one-bead-one-compound combinatorial library to incorporate unnatural amino acids coupled to the negatively charged proteoglycan dermatan sulfate (DS) known to interact with P-selectin (84). DS-IkL localized at regions of vascular inflammation can reduce IS and cTnI levels in a murine IR model. Mice treated with DS-IkL at the onset of reperfusion and additionally 24 h later showed reduced neutrophil extravasation, macrophage accumulation, fibroblast, and endothelial cell proliferation, and fibrosis compared to the non-treated mice.

### Other Inflammation-Inhibiting Peptides

***Ghrelin*** is an octanoylated, 28-mer peptide, which is mainly generated in the stomach and also in small amounts in other organs such as the heart (146). The first evidence of a cardioprotective effect of Ghrelin administered at the onset of reperfusion was obtained in an *ex vivo* rat model of MIRI showing the reduced myocardial release of lactate dehydrogenase (LDH) and myoglobin as well as the depletion of myocardial ATP (85). In a IR mouse model, Ghrelin was reported to reduce IS and inflammation when administered for 3 days before AMI (147). In a more relevant model of MIRI, Wang et al. showed that one-shot Ghrelin administration (8 nmol/kg, i.p.) at the onset of reperfusion protected the rat heart against IR injury by inhibiting oxidative stress and inflammation *via* TLR4/NLRP3 signaling pathway (86).

***Obestatin*** a 23-mer peptide issued from the carboxy-terminal part of proghrelin (ghrelin derives from the amino-terminal part of the same precursor) was reported to protect cardiomyocytes from MIRI *in vitro* and *in vivo* (87, 88, 148). Obestatin administrated by local injection in the left myocardium at the onset of reperfusion was able to reduce IS by ~24% in a rat IR model and to decrease mRNA levels of TNF-α, IL-6, ICAM-1, and iNOS in rat cardiomyocytes after reperfusion (87). Nearly in parallel, Penna and colleagues observed a ~50%–decreased IS after the administration of 75 nM Obestatin during the 20 first min of reperfusion in rats (88).

## Therapeutic Peptides Reducing Necroptosis To Treat MIRI

To our best knowledge, very few peptides were identified as therapeutic treatment inhibiting necroptosis.

The only peptide recently reported is ***Nesfatin-1***; a new energy-regulating peptide displaying a pivotal role in the modulation of cardiovascular functions and protection against MIRI (89). A previous *ex vivo* study on rats revealed that Nesfatin-1 administration in the first 20 min of reperfusion decreases IS by the same extent as ischemic postconditioning through the activation of the pro-survival kinase ERK1/2 (91). In a rat MI model, Nesfatin-1 intraperitoneal injection provided a 50% IS reduction associated with a reduction in Beclin-1 (autophagy) and Caspase-3 (apoptosis) expression. Later on, this group demonstrated that only a high dose of Nesfatin-1 (20 μg/kg) was able to inhibit the expression of RIPK1, RIPK3, MLKL, ROCK1, and ROCK2 proteins (necroptosis and necrosis) in the same rat IR model (90).

## Therapeutic Peptides Reducing Autophagy To Treat MIRI

The anti-apoptotic and pro-angiogenic effects of erythropoietin (EPO) have prompted a growing interest as a therapeutic molecule for the treatment of AMI and HF. Despite promising results in animal MI models where EPO reduces IS and maintains ventricular function (149), clinical studies have revealed controversial results and both safety and tolerability problems.

In 2008, Brines and colleagues designed an 11-mer peptide issued from the helix B of the EPO receptor beta-common chain subunit (= helix B surface peptide, HBSP) (150). *In vivo* studies in MI models have confirmed that HBSP protects the heart from ischemic damage in the same way as EPO (151). Further on, Lin et al. demonstrated that HBSP pretreatment attenuated diabetic cardiomyopathy by inhibiting AMPK-dependent autophagy (152). Another study reported that the protective effect of HBSP against IR injury (i.p. 90 μg/kg, 5 min before reperfusion) is based on its inhibitory effect on cell autophagy (92). Furthermore, HBSP treatment in a hypoxia/reoxygenation-induced apoptosis model on H9c2 cells revealed an inhibition of the autophagy-related proteins (LC3II/LC3I) expression and an enhanced expression of phosphorylated phosphoinositide 3-kinase (PI3K) (153).

## Other Therapeutic Peptides To Treat MIRI

### GLP-1 and GLP-1 Agonists

Glucagon-Like Peptide 1 [GLP-1, also known as GLP-1(7–36)amide] was reported to exert biological actions in the cardiovascular system. Pharmacological postconditioning with GLP-1 has been found effective to reduce IS *in vivo* in rats subjected to IR (94, 154). In isolated mouse hearts, administration of GLP-1(9–36)amide (0.3 nM) induced a 32% IS decrease associated with PI3K-protein Kinase B (PKB)/Akt- and ERK1/2-dependent mechanisms (155).

The first clinical trial (172 patients) evaluating Exenatide, a GLP-1 receptor agonist used as an antidiabetic drug (25 μg/250 mL saline 15 min before and 6 h after reperfusion) revealed promising results in IS reduction (107). Woo et al. showed in addition to IS reduction an improvement of left ventricular function at 6 months post-MI in 58 patients treated by exenatide 20 μg during the percutaneous coronary intervention (PCI) and 10 μg twice daily during 48 h post-MI (108). Roos et al. did not confirm the same cardioprotective effects in their cohort of 191 patients despite a prolonged treatment duration (10 μg/h for 30 min followed by 0.84 μg/h for 72 h) (109). Cardioprotection was observed with another agonist, Liraglutide, showing a reduced necrotic area (156) and improved left ventricular ejection fraction after PCI (157). The mechanisms of action of the GLP-1 receptor agonist modulates myocardial metabolism and hemodynamic effects including peripheral, pulmonary, and coronary vasodilatation, mimicking ischemic preconditioning (158).

A recent clinical trial COMBAT-MI combining remote ischemic conditioning (RIC) and exenatide administration shows that neither RIC nor exenatide, or their combination, were able to reduce IS in STEMI patients when administered as an adjunct to primary percutaneous coronary intervention (110).

### Apelin and Derived Peptides

Apelin (APLN) is the endogenous ligand for the G-protein-coupled apelin receptor (APJ receptor) (159) synthesized as a 77-amino acid prepropeptide further processed into C-terminal fragments Apelin-36, Apelin-19, Apelin-17, and Apelin-13. The adipocytokine Apelin plays a critical role in cardiovascular hemostasis. Secreted in myocardial cells and coronary endothelium, its expression is increased during myocardial damage (160). Since the lack of Apelin was reported to compromise functional recovery of the injured heart, Apelin and its derived peptides were administered as therapeutic molecules. Simpkin et al. demonstrated for the first time that pharmacological postconditioning with Apelin-13 and Apelin-36 peptides protects the heart against IR injury *in vivo* through the RISK pathway activation and by delaying the mPTP opening, resulting in a 43% and a 33%-decreased IS, respectively (161). Additionally, Apelin-16 was shown to increase the contractility of reperfused rat hearts (*ex vivo*) *via* the activation of pro-survival kinases (PKC and ERK1/2) (162).

In obese mice (High-fat diet model), pharmacological postconditioning with Apelin-13 was reported to decrease infarct size, prevent apoptosis and mitochondrial damage induced by IR injury (95). A new endogenous ligand of the Apelin-APJ axis (Elabela also called Toddler or Apela) allowing to protect against IR-induced fibrosis, apoptosis, and oxidative stress *via* the PI3K/AKT signaling pathway has been identified (96).

## Vasoactive Therapeutic Peptides

### Atrial and Brain Natriuretic Peptides

The natriuretic peptide (NP) system consists of at least two distinct endogenous peptides: atrial natriuretic peptide (ANP) and brain (or B-type) natriuretic peptide (BNP). Due to the endocrine function of the heart, these peptide-hormones are secreted inducing specific signals *via* c-GMP coupled receptors. Besides different functions (e.g., lipolysis, lipid oxidation, mitochondrial respiration), NPs play an important role in cardiac vascularization reducing arterial blood pressure as well as sodium reabsorption (163).

NPs were recognized as cardioprotective compounds for MIRI in different animal models based on data showing that ***ANP*** administration reduced IS, increased blood flow, and decreased both left ventricular systolic and end-diastolic pressures in dogs subjected to myocardial IR (70). More recently, IS reduction by ANP was confirmed by Asanuma et al. in a more severe IR dog model (71). Similar results were obtained with ***BNP*** in a rat IR model showing reduced IS and decreased LDH and CK levels compared to untreated animals (74). Thereafter, Li et al. using a recombinant BNP (RhBNP) demonstrated the attenuation of inflammatory infiltration and CD4+ T cell proliferation function in addition to IS reduction (75).

In 2003, hANP treatment was reported to limit IR injury on a small cohort of 19 AMI patients (164). The subsequent study revealed that an ANP infusion during >48 h allows preventing LV remodeling in 50 patients with first anterior AMI (165). Afterward, ANP cardioprotective effects were confirmed in AMI patients by a large multi-center randomized trial (J-WIND—Japan-Working Groups of Acute Myocardial Infarction for the Reduction of Necrotic Damage) (166). Patients treated by human ANP had a reduced IS (-14.7%) assessed by a CK release, an increased LVEF (5.1%), a reduced IR injury (25.9%), and more importantly, decreased risks of cardiac death or HF compared to the control group (103, 166, 167).

In Japan and the US, ***Carperitide*** (28-mer synthetic ANP) or ***Nesiritide*** (23-mer synthetic BNP) have been approved as a treatment for acutely decompensated HF. However, adverse events were observed such as relevant hypotension (102–104) or increased mortality (168) and worsened renal function for Nesiritide (105, 106).

### Derivates of Natriuretic Peptides

#### Urodilatin

Urodilatin an ANP homolog was used as a pharmacological postconditioning in MIRI models. For example, the cyclic 31-mer peptide showed an increased LV pressure in a rat *ex vivo* IR model when applied during the first 15 min of reperfusion and, furthermore, a reduced IS in a pig *in vivo* MIRI model after an intravenous administration during the first 25 min of reperfusion (72).

#### C-type Natriuretic Peptide

C-type natriuretic peptide (CNP) is a 22-mer peptide, structurally related to but genetically distinct from ANP and BNP. Isolated rat hearts subjected to MIRI revealed smaller IS and a reduced coronary perfusion pressure when treated with CNP during the first 30 min of reperfusion (30 nmol/L) (77).

#### Lebetin 2

Lebetin 2 (L2), a 38-mer peptide snake venom-derived NP isolated from *Macrovipera lebetina*, has the advantage to be more stable compared to human NPs. L2 perfused to rat hearts *ex vivo* reduced IS similarly to BNP (73). Interestingly, the same authors showed some years later that L2 has strong and prolonged cardioprotective effects in post-MI (mouse and rat IR models) mainly due to modulation of the inflammatory response as evidenced by enhanced M2-like macrophage detection (76).

#### Vasonatrin Peptide

Vasonatrin peptide (VNP) is an artificial 22-mer chimeric peptide issued from ANP and CNP showing more potent diuretic, natriuretic, and vasorelaxant properties compared with other NPs (169). This peptide was able to attenuate MIRI in diabetic rats (administrated 10 min before R) as demonstrated by reduced LV systolic and end-diastolic pressure as well as decreased Caspase-3 activity and plasma CK/lactate dehydrogenase (LDH) quantities (78).

## Conclusions

Cardiovascular diseases including AMI ranks first in worldwide mortality and according to WHO, they will stay at this rank until 2030. Currently, despite promising results in animal models, there is no pharmacological treatment, which could be administrated in adjunct to reperfusion therapy to inhibit its adverse effects known as reperfusion injury. Differences between preclinical animal MI models and the clinical scenario in patients, including age, comorbidities, and cotreatments could be an explanation (170, 171). Other reasons could be related to the limited comprehension of the underlying pathophysiology and the absence of specific biomarkers to clearly identify MIRI.

Finally, the therapeutic time-window for the application of pharmacological therapies plays a critical role to provide a successful treatment of reperfusion injury. The burst of cell death induced by reperfusion after prolonged ischemia can only be prevented if the administration of cardioprotectants occurs at the onset of reperfusion and preferably before reopening of the culprit coronary artery (26, 172, 173). However, even if the time-window of drug administration should be before reperfusion, further investigations are needed to define how long the protective therapy must be applied to fully prevent MIRI.

## Perspectives

Since MIRI is a complex interplay of different pathways, a strategy involving multiple targets should be considered in the development of pharmacological drugs. Some of the pathways currently being targeted are the different apoptotic pathways, microvasculature circulation, inflammation, platelets, mitochondrial dynamics, and RISK/SAFE pathways leading to cell survival and improved cardiac function (174).

After disappointing results in clinical assays (2014–2016), academic research has forwarded the development of novel therapeutic molecules such as highlighted by more than 1,000 PubMed publications in 2021 (searching keywords: “myocardial ischemia-reperfusion,” “therapy,” “2021”).

To succeed in cardioprotection, other aspects of reperfusion injury besides infarct size should be considered in particular microvascular injury since heart function is ensured by both cardiomyocytes and vascular cells (cell ratio 50:50) (174) and early ventricular arrhythmias with a lower contribution.

Additionally, considering the development of new therapeutic peptides (or other pharmacological drugs), special attention should be devoted to the improvement of targeting the ischemic zone (or subcellular localization) within the infarcted heart to maximize local drug concentration and reduce side effects. Recently, Zhang and co-workers showed higher mitochondrial integrity, lower apoptosis of cardiomyocytes, and reduced myocardial IS by encapsulating CsA in PEGylated nanoparticles with mitochondria-targeting [CsA@PLGA-PEG-SS31] (127). Another example is the cyclic heart homing sequence [CSTSMLKAC] grafted on porous silicon nanoparticles revealing an improved accumulation within the heart (up to three-fold) (175).

Taking together, future development of pharmacological drugs to treat AMI patients should be characterized by a drug cocktail or a pleiotropic drug acting specifically on (i) different pathways or (ii) different cell types, or by (iii) an improved tissue or subcellular targeting. The combination of these strategies should provide advantages for future clinical outcomes.

## Author Contributions

PB and SB-L contributed to conception and design of the manuscript. CFR, KK, and PB performed the selection of the therapeutic peptides. PB wrote the first draft of the manuscript. CFR, KK, EJ, JN, SB-L, and PB wrote sections of the manuscript. EJ performed the graphical design of the figures. All authors contributed to manuscript revision, read, and approved the submitted version.

## Funding

ANR grants for the LabEx ICST [ANR-11-LABX-0015] and for HFADD [ANR-17-CE18 0007]—Grants Fondation de France (AAP 2019, #00096298), Pré-maturation 2017 Région Occitanie (PepCard, ESR_PREMAT-000019), and French Ministry for a PhD fellowship.

## Conflict of Interest

The authors declare that the research was conducted in the absence of any commercial or financial relationships that could be construed as a potential conflict of interest.

## Publisher's Note

All claims expressed in this article are solely those of the authors and do not necessarily represent those of their affiliated organizations, or those of the publisher, the editors and the reviewers. Any product that may be evaluated in this article, or claim that may be made by its manufacturer, is not guaranteed or endorsed by the publisher.
